# A novel NF-κB/YY1/microRNA-10a regulatory circuit in fibroblast-like synoviocytes regulates inflammation in rheumatoid arthritis

**DOI:** 10.1038/srep20059

**Published:** 2016-01-29

**Authors:** Nan Mu, Jintao Gu, Tonglie Huang, Cun Zhang, Zhen Shu, Meng Li, Qiang Hao, Weina Li, Wangqian Zhang, Jinkang Zhao, Yong Zhang, Luyu Huang, Shuning Wang, Xiaohang Jin, Xiaochang Xue, Wei Zhang, Yingqi Zhang

**Affiliations:** 1 State Key Laboratory of Cancer Biology, Department of Biopharmaceutics, School of Pharmacy, Fourth Military Medical University, Xi’an, China; 2 Department of Pharmacogenomics, School of Pharmacy, Fourth Military Medical University, Xi’an, China; 3 Department of Clinical Immunology and Rheumatology, Xijing Hospital, Fourth Military Medical University, Xi’an, China; 4 Institute of Orthopedics, Tangdu Hospital, Fourth Military Medical University, Xi’an, China; 5 Department of Orthopedics, Xijing Hospital, Fourth Military Medical University, Xi’an, China; 6 Department of Human Anatomy, Histology, and Embryology, Fourth Military Medical University, Xi’an, China

## Abstract

The main etiopathogenesis of rheumatoid arthritis (RA) is overexpressed inflammatory cytokines and tissue injury mediated by persistent NF-κB activation. MicroRNAs widely participate in the regulation of target gene expression and play important roles in various diseases. Here, we explored the mechanisms of microRNAs in RA. We found that microRNA (miR)-10a was downregulated in the fibroblast-like synoviocytes (FLSs) of RA patients compared with osteoarthritis (OA) controls, and this downregulation could be triggered by TNF-α and IL-1β in an NF-κB-dependent manner through promoting the expression of the YingYang 1 (YY1) transcription factor. Downregulated miR-10a could accelerate IκB degradation and NF-κB activation by targeting IRAK4, TAK1 and BTRC. This miR-10a-mediated NF-κB activation then significantly promoted the production of various inflammatory cytokines, including TNF-α, IL-1β, IL-6, IL-8, and MCP-1, and matrix metalloproteinase (MMP)-1 and MMP-13. In addition, transfection of a miR-10a inhibitor accelerated the proliferation and migration of FLSs. Collectively, our data demonstrates the existence of a novel NF-κB/YY1/miR-10a/NF-κB regulatory circuit that promotes the excessive secretion of NF-κB-mediated inflammatory cytokines and the proliferation and migration of RA FLSs. Thus, miR-10a acts as a switch to control this regulatory circuit and may serve as a diagnostic and therapeutic target for RA treatment.

Rheumatoid arthritis (RA) is an autoimmune disease in which fibroblast-like synoviocytes (FLSs), a specialized cell type located in synovial joints, play crucial roles in the damage, destruction and deformation of cartilage and joints. The pathologic remodeling of the cartilage, tendons and bone associated with the RA process can mainly be ascribed to the effects mediated by FLSs^1^,^2^. During the progression of RA, constant inflammatory responses occur in the synovial membrane; the proliferative and apoptotic properties of FLSs are then changed, and the cell number is greatly increased. These cells, together with other immune cells, including macrophages, dendritic cells, lymphocytes, mast cells and platelets, can disrupt immune homeostasis and create an inflammatory environment in the synovium, which attracts more immune cells and, thus, eventually contributes to cartilage damage and joint destruction^3^,^4^. In addition, FLSs can promote various processes in RA by secreting different types of inflammatory cytokines, such as IL-6, IL-8, IL-1β, TNF-α, and MCP-1, and matrix metalloproteinases (MMPs), such as MMP-1 and MMP-13^5^,^6^,^7^,^8^.

MicroRNAs (miRNAs) are evolutionarily conserved small non-coding RNAs that are 19–25 nucleotides in length and post-transcriptionally modulate the expression of downstream target genes by repressing translation or accelerating mRNA degradation^9^,^10^. Thousands of unique mature miRNAs have been identified in different species, and approximately 1,800 miRNAs are known to be expressed in human cells (http://microrna.sanger.ac.uk). A single miRNA can target hundreds of mRNAs, and multiple miRNAs can regulate a specific gene, facilitating the possibility of finely tuning gene expression. The inappropriate production of miRNAs has generally been regarded as a major feature of a wide spectrum of human diseases, including developmental abnormalities, cancer, and autoimmune diseases such as multiple sclerosis, RA and inflammatory bowel diseases^11^,^12^,^13^,^14^,^15^,^16^,^17^. For example, let-7e is upregulated in experimental autoimmune encephalomyelitis (EAE), and silencing let-7e *in vivo* inhibits encephalitogenic Th1 and Th17 cells and attenuates EAE; by contrast, let-7e overexpression enhances Th1 and Th17 cells and aggravates EAE^18^. miR-146a can orchestrate inflammatory responses in murine inflammatory bowel disease by activating hedgehog signaling^19^.

Recently, several miRNAs have been found to be dysregulated in RA patients^20^,^21^,^22^,^23^. Kawano and colleagues reported that miR-124 ameliorated adjuvant-induced arthritis (AIA) by suppressing RANKL and NFATc1, making miR-124 a candidate for the treatment of human RA^24^. miR-30a-3p could play a critical role in the autoimmune responses that occur in RA by regulating B cell-activating factor (BAFF) expression^25^. However, the detailed molecular mechanisms underlying how the dysregulated miRNAs affect RA progression remain to be elucidated.

Using a miRNA microarray, we compared the miRNA expression profiles of the FLSs of RA and osteoarthritis (OA) patients. Approximately 380 miRNAs were found to be differentially expressed. Among these miRNAs, miR-10a was highly expressed in OA but showed very low levels in RA patients. In addition, miR-10a was further downregulated in TNF-α-treated FLSs. Our preliminary data indicated that the repression of miR-10a by TNF-α is NF-κB-dependent. When miRNA target-detection programs were used to predict the candidate target genes of miR-10a, several NF-κB activation-associated genes, such as interleukin-1 receptor-associated kinase 4 (IRAK4), TGF-beta-activated kinase 1 (TAK1) and β-transducing repeat-containing protein 1 (BTRC), were predicted to be candidate target genes of miR-10a. Therefore, we predict that a novel TNF-α/NF-κB/miR-10a/NF-κB regulatory circuit exists in FLSs, which contributes to the exaggerated activation of the NF-κB signaling pathway and plays a critical role in inflammatory responses in RA. The aim of this study was to verify the detailed molecular mechanism of miR-10a in this regulatory circuit and, thus, provide a novel potential therapeutic target for the treatment of RA.

## Results

### miR-10a is downregulated in synovial tissues of RA patients

**Figure 1:**
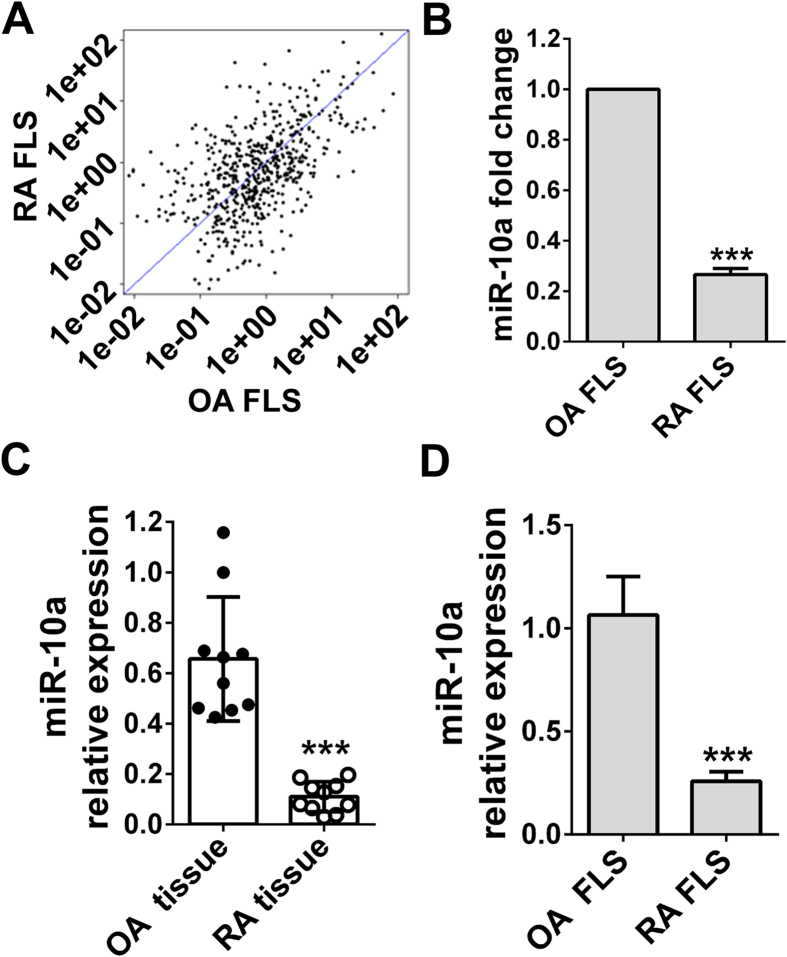
miR-10a is downregulated in the synovial tissues and FLSs of RA patients. **(A)** Total RNA was isolated from FLSs that were isolated from RA and OA patients and subjected to microRNA microarray analysis on the Lianchuan human genome microRNA array. The miRNA expression profiling data are shown with scatter plots. One representative of three experiments is shown. The top ten differentially expressed miRNAs in RA FLSs are shown in a heat map diagram (supplementary Fig. S1). **(B)** The miR-10a levels detected using the microarray were normalized to a negative control of random sequences of similar sizes. **(C)** Quantitative PCR validation of the microRNA microarray. Synovial tissues were collected from RA (n = 10) and OA (n = 10) patients. Total mRNA was extracted, and the miR-10a expression level was analyzed through quantitative real-time PCR and normalized to the U6B housekeeping microRNA gene. **(D)** miR-10a expression was downregulated in the FLSs of RA patients compared with OA patients. ***P < 0.001 compared with the OA group. The results are expressed as percentages of the control mean values ± SD for three independent experiments.

Although accumulating evidence suggests that miRNAs play critical roles in various human diseases, such as cancer^16^, developmental abnormalities, and muscular and cardiovascular disorders^26^, the role and detailed mechanism of miRNAs in RA is still far from clear. To determine whether RA patients exhibit a unique miRNA expression profile, total RNAs were isolated from the FLSs of RA and OA patients, and miRNA microarray profiling of human miRNA genes was performed (Supplementary Fig. S1). Approximately 380 miRNAs were differentially expressed between the FLSs of RA and OA patients (Fig. 1A), among which miR-10a was highly expressed in OA patients but showed very low levels in RA patients (Fig. 1B, *P* < 0.001). To confirm this finding, synovial tissues and primary FLSs were isolated from RA and OA patients (n = 10 for each), and miR-10a levels were detected via real-time PCR. As expected, miR-10a expression was downregulated approximately 4–5-fold in the synovial tissues and FLSs of RA patients (Fig. 1C, D).

### miR-10a is downregulated by TNF-α and IL-1β in an NF-κB-dependent manner in RA FLSs

**Figure 2:**
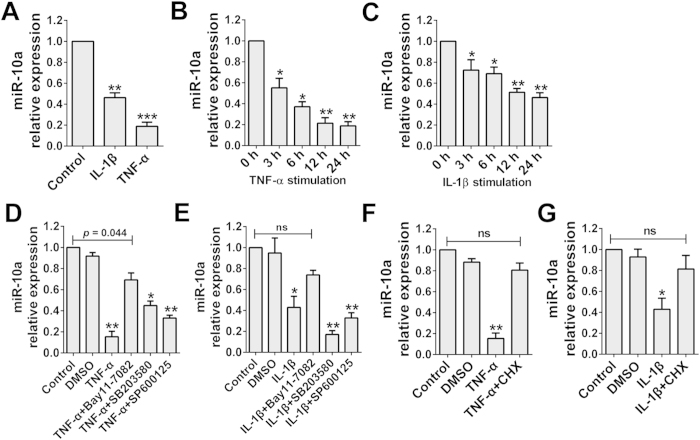
miR-10a is downregulated by TNF-α and IL-1β in an NF-κB-dependent manner. **(A)** RA FLSs (n = 3 samples) were stimulated with TNF-α or IL-1β (10 ng/ml) for 24 h, and the miR-10a expression level was analyzed through real-time PCR. U6B was used as the negative control. **(B)** RA FLSs (n = 3 samples) were treated with TNF-α for the indicated period, and the miR-10a expression level was detected via real-time PCR. **(C)** Cells were treated with IL-1β, and miR-10a was detected as shown in **(B)**. **(D)** Cells were treated with TNF-α either alone or in the presence of inhibitors of NF-κB (Bay 11-7082), JNK (SP600125) or p38MAPK (SB203580), and miR-10a was detected 24 h later. **(E)** The same method was applied as in **(D)**, except that TNF-α was replaced by IL-1β. **(F)** TNF-α-stimulated cells were pretreated with CHX, and the miR-10a expression level was analyzed 24 h later through real-time PCR. **(G)** The same method was applied as in **(F)**, except that TNF-α was replaced with IL-1β. *P < 0.05, **P  < 0.01 or ***P < 0.001 compared with the control group. Data are representative of three experiments.

To investigate how miR-10a is downregulated in RA FLSs, the classical proinflammatory factors TNF-α and IL-1β, which have been employed clinically as therapeutic targets in RA treatment, were used to treat FLSs. The results showed that miR-10a was significantly downregulated by both TNF-α and IL-1β (Fig. 2A). Moreover, the downregulation of miR-10a by TNF-α and IL-1β was time dependent (Fig. 2B,C.)

It is well known that the functions of both TNF-α and IL-1β are mainly mediated by activation of the NF-κB, JNK or p38/MAPK signaling pathway. To determine whether these pathways are involved in the downregulation of miR-10a in FLSs, we treated FLS cells with TNF-α and IL-1β in the presence of inhibitors of NF-κB (Bay 11–7082), JNK (SP600125) or MAPK (SB203580). As shown in Fig. 2D,E, blockade of NF-κB activation by Bay 11–7082 notably reversed the repression of miR-10a by TNF-α and IL-1β. However, neither the JNK nor the MAPK inhibitor had a significant effect on miR-10a expression. These data suggested that TNF-α and IL-1β downregulate FLS miR-10a expression in an NF-κB-dependent and JNK- and MAPK-independent manner.

We further determined whether miR-10a is regulated directly by NF-κB. Cycloheximide (CHX), a protein synthesis inhibitor, was added to RA FLSs, which were then stimulated by TNF-α or IL-1β. The results showed that CHX potently impaired the regulation of miR-10a by TNF-α and IL-1β (Fig. 2F,G). This finding indicated that NF-κB regulates miR-10a indirectly and that *de novo* synthesis of new protein is indispensable for miR-10a repression.

### miR-10a is directly downregulated by YY1

**Figure 3:**
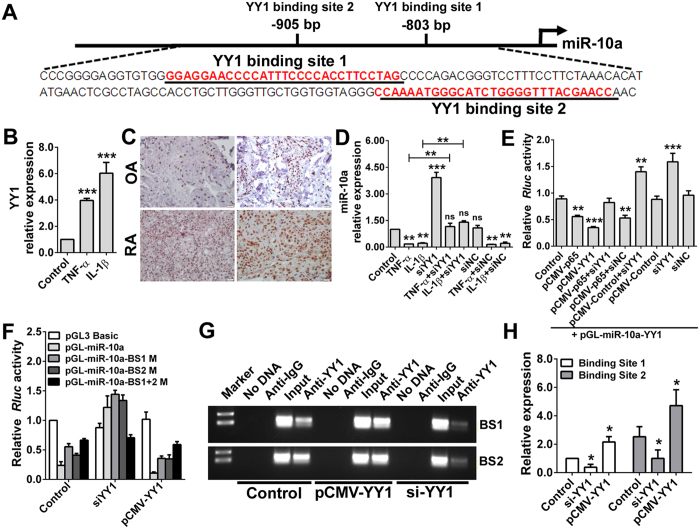
miR-10a is directly downregulated by YY1. **(A)** Schematic diagram showing the promoter sequence of miR-10a, which contains two YY1 binding sites. **(B)** RA FLS (n = 3 samples) primary cells were stimulated with TNF-α or IL-1β for 24 h, and the YY1 level was detected via real-time PCR. **(C)** The YY1 expression level was detected in synovial tissue specimens from RA and OA patients (n = 5 samples) through immunohistochemistry. **(D)** RA FLSs (n = 3 samples) were treated with TNF-α or IL-1β, with or without interference with YY1 (siYY1), and the miR-10a expression level was detected via qRT-PCR. **(E)** HEK293T cells were transfected with a pGL-miR-10a-YY1 luciferase reporter vector constructed with the promoter region of miR-10a that contains the two YY1 binding sites, either alone or in combination with pCMV-p65, pCMV-YY1 or siYY1. Groups subjected to treatment with pGL-miR-10a-YY1 alone, scramble siRNA (siNC) or the empty vector (pCMV-control) were used as control groups. *Renilla* luciferase activity was measured 24 h later. **(F)** HEK293T cells were transfected with luciferase reporter vectors constructed with the miR-10a promoter region containing mutations in YY1 binding site 1 (pGL-miR-10a-BS1 M) or binding site 2 (pGL-miR-10a-BS2 M), or in both sites (pGL-miR-10a-BS1+2 M), and luciferase activity was measured 24 h later. **(G)** HEK293T cells were transfected with pCMV-YY1 or siYY1 to increase or reduce YY1 levels, and a ChIP assay was used to detect the direct binding of YY1 to the miR-10a promoter. **(H)** Statistical analysis of (G). ^ns^*P* > 0.05,*P < 0.05, **P < 0.01 or ***P < 0.001 compared with the control group. Data are representative of three experiments.

Considering that newly synthesized protein was found to directly participate in miR-10a regulation, we next analyzed the promoter region of miR-10a, and several transcription factor binding sites were found. Among these sites, there were two binding sites in the miR-10a promoter for YY1, which is a ubiquitously distributed transcription factor involved in repressing diverse promoters and was selected for further study (Fig. 3A). As shown in Fig. 3B, both TNF-α and IL-1β stimulation dramatically upregulated the transcription of YY1 in RA FLS cells compared with untreated FLS cells (fold change >4 and *P* < 0.001). Immunohistochemistry results also demonstrated that YY1 was overexpressed in the synovial tissue of RA patients but presented much lower levels in OA patients (Fig. 3C). These data suggested that YY1, which is stimulated by NF-κB activation, may mediate the downregulation of miR-10a in RA patients.

To verify that YY1 is the critical factor downstream of TNF-α and IL-1β for miR-10a regulation, FLS cells were treated with TNF-α or IL-1β in the presence of YY1 siRNA. As shown in Fig. 3D, approximately 4-fold overexpression of miR-10a was observed in YY1-knockdown RA FLSs compared with the control cells (*P* < 0.001). In addition, knockdown of YY1 almost completely abrogated the regulation of miR-10a by TNF-α and IL-1β. Additionally, a luciferase reporter assay was performed, and the results showed that YY1 induced reproducible significant miR-10a promoter-driven repression of the luciferase reporter when ectopically expressed in HEK293T cells (Fig. 3E). This is a unique feature of YY1 as mutation of the two YY1 binding sites in the promoter of miR-10a effectively shielded this phenomenon. That is, neither the silencing of YY1 (siYY1) nor the ectopic expression of YY1 (pCMV-YY1) had an effect on the *Renilla* luciferase-containing mutated miR-10a promoter (Fig. 3F). To further explore the binding of the miR-10a promoter by YY1 under physiological conditions, a ChIP assay was performed, and the results revealed that YY1 could directly bind the promoter of miR-10a and repress its expression in HEK293T cells (Fig. 3G,H). Taken together, these experiments firmly established miR-10a as an YY1-specific target gene in RA FLSs.

**Figure 4:**
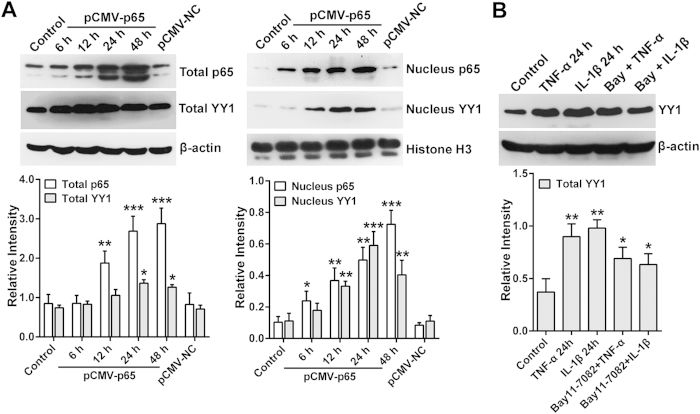
TNF-α and IL-1β or activated p65 promotes YY1 expression and translocation. **(A)** HEK293T cells were transfected with pCMV-p65, and the expression levels of p65 and YY1 were detected by Western blotting. **(B)** RA FLSs were stimulated with TNF-α or IL-1β, either alone or in combination with Bay 11–7082 for 24 h, and the YY1 level was detected by Western blotting. The data are representative of three experiments (Supplementary Fig. S3).

As YY1 plays a vital role in miR-10a regulation, we further examined whether TNF-α- and IL-1β-activated NF-κB can promote YY1 protein expression. HEK293T cells were transfected with pCMV-p65, and the YY1 expression level was detected by Western blotting. As shown in Fig. 4A, p65 indeed enhanced the expression and nuclear distribution of YY1 in HEK293T cells. In addition, both TNF-α and IL-1β stimulation promoted YY1 expression in RA FLSs, and this effect could be inhibited to some extent by Bay 11–7082 (Fig. 4B). These data further demonstrate that YY1 harbors highly potent and specific activity to mediate the TNF-α and IL-1β repression of miR-10a expression.

### IRAK4, TAK1 and BTRC are target genes of miR-10a

miRNAs mainly function as negative regulators of target genes by binding to the 3′-UTRs of these genes and accelerating mRNA degradation or blocking protein expression. To investigate the pathological role of miR-10a downregulation in RA, miRNA target-detection procedures were used to predict the candidate target genes of miR-10a. Interestingly, several genes, including IRAK4, TAK1 and BTRC, which participate in the NF-κB signaling pathway, were predicted to be candidate target genes of miR-10a through various miRNA target gene prediction procedures.

**Figure 5:**
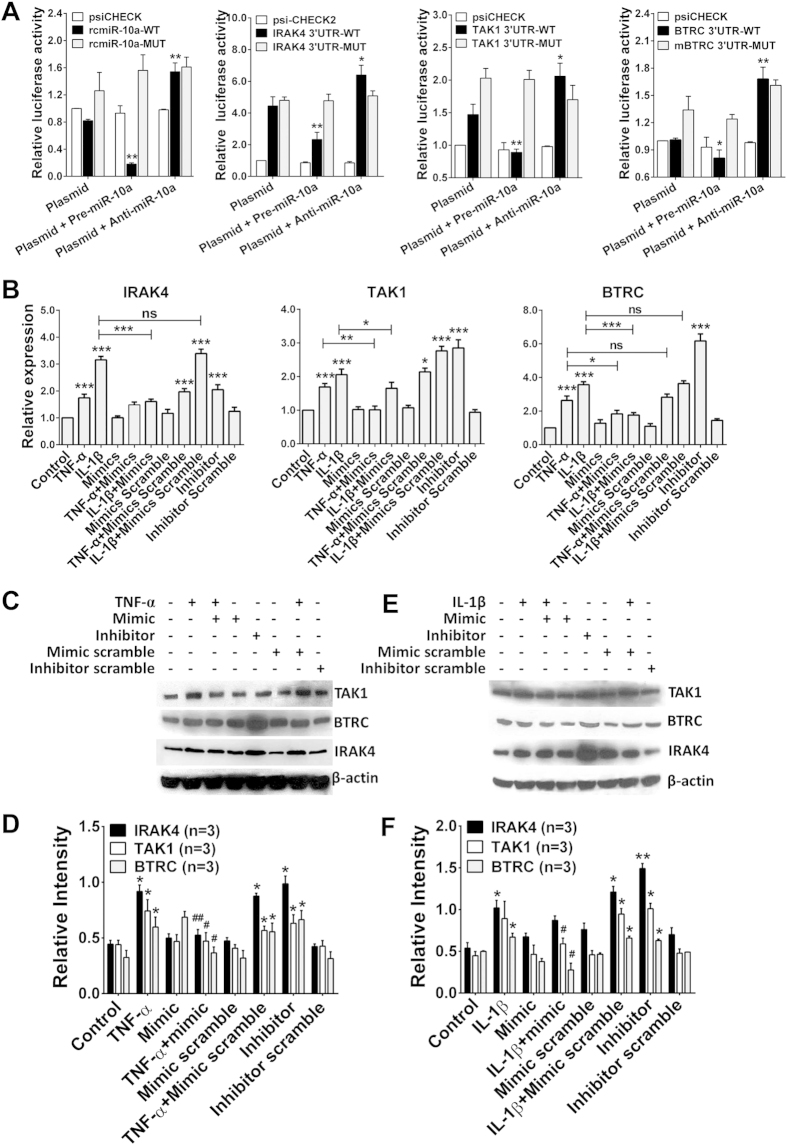
IRAK4, TAK1 and BTRC are target genes of miR-10a. **(A)** Dual-luciferase reporter assays of rcmiR-10a/mrcmiR-10a, IRAK4/mIRAK4, TAK1/mTAK1 or BTRC/mBTRC, either alone or in the presence of miR-10a precursors or inhibitors. Vectors containing rcmiR-10a and mrcmiR-10a were used as positive controls. The decrease in *RLuc* was measured and normalized to FLuc activity, and the constructed vector was normalized to an empty psi-CHECK-2 vector. *P < 0.05, **P < 0.01 compared with the plasmid-only group. **(B)** FLS cells (n = 3 samples, same as in Fig. 5C, E) were treated as indicated, and IRAK4, TAK1 and BTRC mRNA levels were detected through real-time PCR. *P < 0.05, **P < 0.01, ***P < 0.001 compared with the control group or between the indicated groups. **(C)** FLS cells were treated with TNF-α in the presence of miR-10a mimics or inhibitors, and IRAK4, TAK1 and BTRC expression levels were detected via Western blotting. **(D)** Statistical analysis of **(C)**. **(E)** FLS cells were treated with IL-1β in the presence of miR-10a mimics or inhibitors, and IRAK4, TAK1 and BTRC expression levels were detected through Western blotting. **(F)** Statistical analysis of **(E)**. *P < 0.05, **P < 0.01, ***P < 0.001 compared with the control group or between the indicated groups.

IRAK4 is a Ser/Thr protein kinase that plays an important role as a signaling mediator in signal transduction facilitated by the Toll-like receptor (TLR) and interleukin-1 receptor (IL-1R) families. TAK1 and BTRC are two negative factors controlling IκB degradation in the type 1 TNF receptor (TNF-RI) and IL-1R pathways upon TNF-α and IL-1β stimulation. To determine whether IRAK4, TAK1 and BTRC are indeed target genes of miR-10a in FLSs, we constructed dual-luciferase reporter vectors containing the predicted seed sequence in the 3′-UTRs of IRAK4, TAK1 and BTRC as well as corresponding mutant vectors in which 3-nt mutations were introduced (Supplementary Fig. S2). All these vectors were used to transfect the HEK293T cell line, either alone or in the presence of a miR-10a precursor or inhibitor, and luciferase activity was analyzed 24 h later. As shown in Fig. 5A, co-transfection of the miR-10a precursor significantly repressed the activity of *RLuc* containing the seed sequences in the 3′-UTRs of IRAK4, TAK1 (*P* < 0.01) and BTRC (*P* < 0.05) compared with the vector-only group. However, transfection of the miR-10a inhibitor upregulated *RLuc* activity (*P* < 0.05). In addition, when the mutant constructs were used, all the effects of the miR-10a precursor and inhibitor disappeared, and no significant differences in *RLuc* activity were found between the different treated groups. All these data indicated that miR-10a specifically targets IRAK4, TAK1 and BTRC.

We then further determined the physiological and pathological roles of miR-10a in the production of IRAK4, TAK1 and BTRC by RA FLSs. FLS cells were treated with the indicated reagents, and target gene mRNA and proteins levels were detected. The results showed that TNF-α or IL-1β stimulation dramatically upregulated IRAK4, TAK1 and BTRC transcription (Fig. 5B). Notably, the mRNA levels of these genes induced by the miR-10a inhibitor were comparable to, if not more potent than, those induced by TNF-α and IL-1β treatment. When TNF-α or IL-1β was used in combination with miR-10a mimics, the enforced expression of miR-10a almost completely reversed the effect and inhibited gene transcription. Finally, Western blotting was used to detect the protein production of these genes and the data were consistent with the observed mRNA levels (Fig. 5C–F). Collectively, these findings indicated that IRAK4, TAK1 and BTRC are specifically regulated by miR-10a in RA FLS cells, and TNF-α and IL-1β regulate these molecules at least partly via repressing miR-10a.

### miR-10a downregulation promotes NF-κB activation and the production of downstream inflammatory cytokines

**Figure 6:**
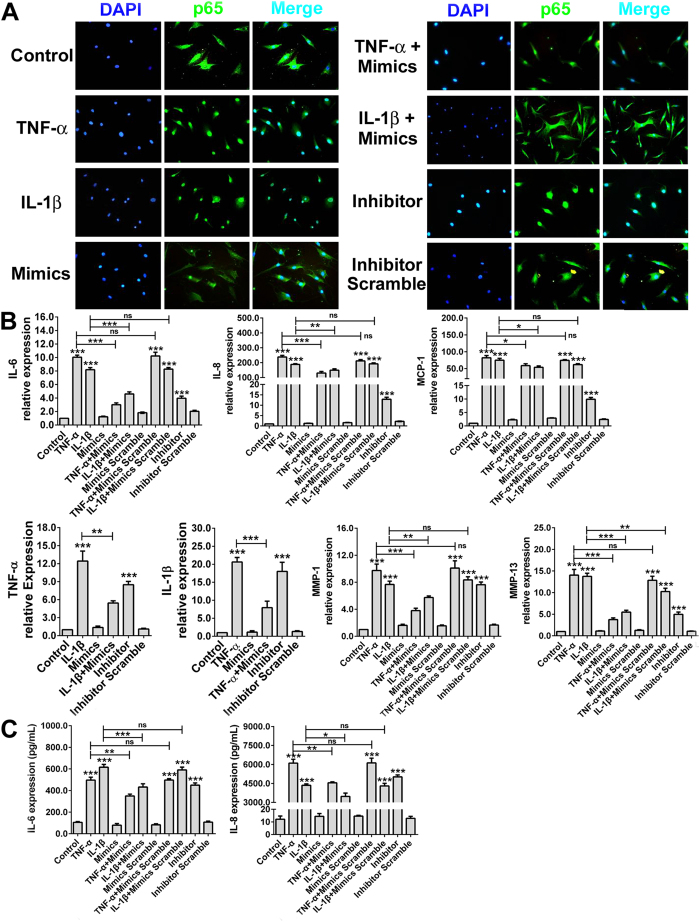
miR-10a repression promotes NF-κB activation and secretion of downstream inflammatory cytokines in FLS cells. **(A)** FLS cells were stimulated as indicated, and an immunofluorescence assay was performed 24 h later to detect the activation and translocation of NF-κB p65. **(B)** FLS cells (n = 3 samples, same as in Fig. 6C) were treated as indicated for 24 h, and the mRNA levels of inflammatory cytokines or MMPs were measured by qRT-PCR. **(C)** Cells were treated as indicated, and IL-6 and IL-8 production was determined by ELISA. ^ns^*P* > 0.05, *P < 0.05, **P < 0.01, ***P < 0.001 compared with the control group or between the indicated groups. The data are representative of three experiments.

We postulated that miR-10a can promote NF-κB activation and translocation by repressing the aforementioned target genes. To confirm this hypothesis, FLS cells were transfected with a miR-10a inhibitor, and immunofluorescence staining was performed to detect NF-κB p65 localization. The results showed that the downregulation of miR-10a by the inhibitor accelerated NF-κB activation and translocation, whereas forced expression of miR-10a with mimics blocked the activation of NF-κB by TNF-α or IL-1β (Fig. 6A).

As miR-10a can effectively promote NF-κB activation and translocation, we next detected the cytokines regulated by NF-κB in FLSs. As shown in Fig. 6B, the expression of several inflammatory cytokines, including IL-6, IL-8, MCP-1, TNF-α and IL-1β, was significantly upregulated by both TNF-α and IL-1β (*P* < 0.05 for MCP-1 and *P* < 0.001 for all the others); this regulation was thoroughly shielded by transfection with miR-10a mimics. Notably, the production of these cytokines was also greatly increased in miR-10a inhibitor-transfected cells. In addition, some MMPs, including MMP-1 and MMP-13, were upregulated as well (Fig. 6B). The protein levels of IL-6 and IL-8 were further confirmed by ELISA (Fig. 6C).

### miR-10a mediates the TNF-α and IL-1β regulation of FLS proliferation, migration and invasion

**Figure 7:**
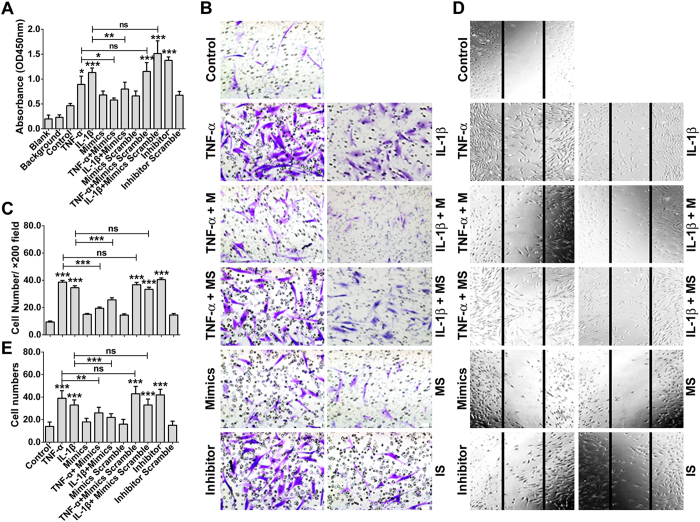
miR-10a mediates the regulation of RA FLS proliferation, migration and invasion by TNF-α and IL-1β. **(A)** RA FLS cells were transfected or treated as indicated, and BrdU was added to the cells, followed by incubation for 24 h. The incorporation of BrdU into newly synthesized cellular DNA was then detected with a POD-conjugated anti-BrdU antibody in a scanning spectrophotometer. Scramble miRNA was used as the negative control. **(B)** FLS cells were treated as indicated, and the cell scratch assay was performed to evaluate the migration ability of the cells. **(C)** Statistical analysis of **(B)**. **(D)** FLS cells were transfected or treated as indicated, and a transwell assay was used to evaluate the effect of miR-10a on the invasion of the cells. **(E)** Statistical analysis of **(D)**. ^ns^*P* > 0.05, *P < 0.05, **P < 0.01, ***P < 0.001 compared with the control group or between the indicated groups. M: miR-10a mimics, MS: mimics scramble, IS: inhibitor scramble. The data are representative of three experiments.

Although it is well known that the irreversible dysregulation of the proliferation, migration and invasion of FLS cells (which can be effectively adjusted by the TNF-α and IL-1β neutralizing antibodies that are used clinically) plays critical roles in RA, the detailed mechanisms are still far from clear. Based on the aforementioned data, we speculated that miR-10a mediated the effects of TNF-α and IL-1β on RA FLSs. To verify this hypothesis, the proliferation, migration and invasion of FLS cells were observed after treatment with TNF-α or IL-1β, either alone or in the presence of miR-10a mimics or a miR-10a inhibitor. As shown in Fig. 7A, treatment with a TNF-α, IL-1β or miR-10a inhibitor enhanced FLS proliferation, whereas the miR-10a mimics effectively weakened the effect of TNF-α and IL-1β. Similar results were obtained in Transwell assays (Fig. 7B,C) and wound closure assays (Fig. 7D,E), in which treatment with TNF-α, IL-1β and miR-10a inhibitors markedly promoted the migration and invasion of FLSs, whereas miR-10a mimics weakened these abilities.

## Discussion

TNF-α and IL-1β are pivotal proinflammatory cytokines that mediate the systemic inflammatory response and regulate immune functions. Inappropriate production of TNF-α and IL-1β and sustained activation of the NF-κB signaling pathway have been implicated in the pathogenesis of a wide variety of human diseases, including diabetes^27^, cancer^28^, osteoporosis, and autoimmune diseases such as RA and inflammatory bowel disease^29^. It is well known that TNF-α and IL-1β and their receptors TNFR1 and IL-1R are abundant in the RA synovium and are necessary for the initiation of chronic and persistent inflammatory responses in RA^30^.

As one of the main cell types located in the inner layer of the synovium, FLSs are the main source of synovial fluid and are important in maintaining the homeostasis of the internal joints. In RA patients, FLSs obtain hyperplastic, inflammatory, and cartilage- and bone-destructive phenotypes. When TNF-α and IL-1β trigger constant inflammatory processes in the synovium, the total number of FLSs increases significantly. These FLS cells contribute to the inflammatory microenvironment and recruit and activate more immune cells to the damaged area by secreting various proinflammatory cytokines and chemokines, especially Il-6, IL-8, and MMPs, and thereby contribute to cartilage damage and joint destruction^31^,^32^.

It is well known that TNF-α- and IL-1β-activated NF-κB is essential for the production of cytokines by RA FLSs and mediates the resistance of RA FLSs to apoptosis^33^,^34^. A recent study found that in addition to acting as a major driver of bone destruction in arthritis, TNF-α can also upregulate the Wnt antagonist dickkopf-1 (Dkk-1) in FLSs, thereby inhibiting new bone formation in RA patients^35^. Therefore, reducing the biological activities of these cytokines is a representative method for treating diseases mediated by proinflammatory cytokines^36^,^37^. All these factors have been demonstrated through the widespread clinical use of TNF-α-neutralizing agents to treat RA patients to improve arthritis and inhibit bone destruction.

Despite these findings, single-cytokine inhibition of TNF-α or IL-1β produces clinically meaningful responses in only approximately half of RA patients^38^,^39^. Therefore, establishing the detailed mechanisms underlying the exaggerated expression of IL-1β and TNF-α and the sustained activation of NF-κB is meaningful.

In recent years, the findings of miRNAs have shed new lights on our understanding of the regulation of gene expression and the pathogenesis of various diseases. Significant abnormal miRNA expression profiles have been reported in RA^40^,^41^. And dozens of miRNAs, such as miR-146a, miR-155 and miR-124a^42^,^43^, has been proved to have essential functions in RA pathological processes including systemic inflammation and joint destruction^44^.

Considering the differences of population and the version of miRNA database may affect miRNA expression profile data, we performed miRNA microarray in RA and OA patients, and miR-10a was found to be 3-fold lower in RA versus OA patients. miR-10a has been found to be an important miRNA in autoimmune diseases, and it was reported previously that miR-10a decreased expression in lupus and inflammatory bowel disease (IBD)^45^,^46^,^47^. In IBD patients, miR-10a contributed to the maintenance of intestinal homeostasis by targeting IL-12/IL-23p40 expression^46^, and is involved in Th1/Th17 cell immune response^47^. But no studies have shown the function of miR-10a in RA.

In this study, we found that the proinflammatory factors TNF-α and IL-1β can downregulate miR-10a in an NF-κB-dependent manner by promoting the production of the transcription factor YY1, a downstream gene of NF-κB. Reduced miR-10a levels in FLSs conversely promote NF-κB activation and translocation by targeting the genes IRAK4, TAK1 and BTRC. Silencing of miR-10a in FLSs enhanced the excessive secretion of inflammatory cytokines, including IL-1β, IL-6, IL-8, TNF-α and MCP-1, and MMPs, such as MMP-1 and MMP-13. TNF-α, IL-1β and IL-6 are strong pathological factors that play a critical role in the onset and progression of RA^48^,^49^. MCP-1 and IL-8 are important chemoattractants for neutrophils, monocytes, memory T cells and dendritic cells, and their induction by miR-10a suggests a link between the innate and adaptive immune responses and the early recruitment of inflammatory cells to the sites of inflammation^50^,^51^. By regulating these cytokines, miR-10a plays a critical role in the onset and development of RA. We also found that downregulation of miR-10a promotes the proliferation, migration and invasion of RA FLSs. Thus, our data reveal a novel TNF-α/NF-κB/YY1/miR-10a/NF-κB regulatory circuit in RA FLSs that contributes to the autoamplification of inflammatory responses of RA, thus providing a novel potential diagnostic and therapeutic target for the treatment of RA.

## Conclusion

**Figure 8:**
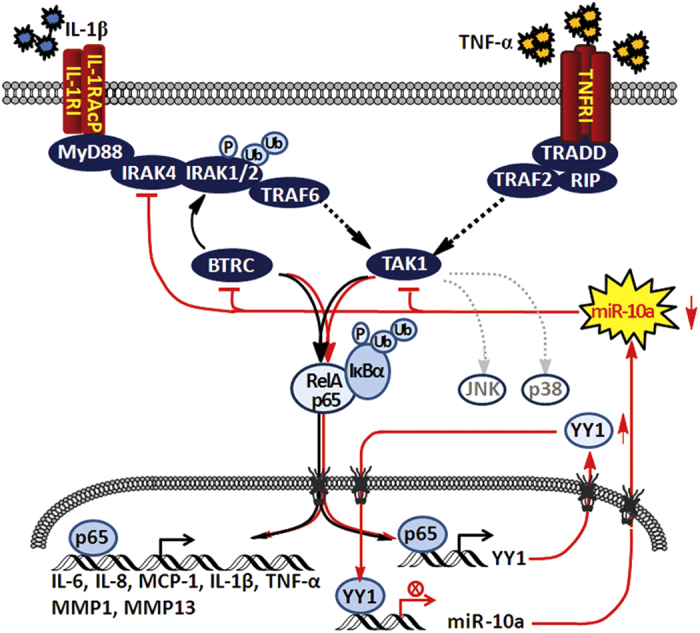
The schematic diagram of the regulatory circuit signaling events involving miR-10a in RA FLSs. NF-κB is liberated and enters the nucleus of RA FLSs when stimulated with TNF-α or IL-1β. Then, NF-κB is activated and binds to the promoter of YY1 and enhances its expression. The latter protein can act as a transcription factor and repress miR-10a expression by binding to the two YY1-binding sites located in the promoter region of miR-10a. Downregulated miR-10a subsequently promotes NF-κB activation and translocation by targeting IRAK4, TAK1 and BTRC. The activated NF-κB then further downregulates miR-10a via YY1. At the same time, this regulatory circuit promotes the release of various inflammatory factors, such as TNF-α, IL-1β, IL-6, IL-8, and MCP-1, and the metalloproteinases MMP-1 and MMP13, which are controlled by NF-κB and accelerate the proliferation, migration and invasion of FLSs in RA. The black lines indicate the canonical NF-κB pathway; the red lines indicate the regulatory circuit reported here.

Taken together, our data explain the redundancy of TNF-α and IL-1β production and NF-κB activation in RA patients and have important implications for our understanding of the maintenance of inflammatory responses in RA. miR-10a is one of the factors that controls the production of inflammatory cytokines and the proliferation and migration of FLSs (Fig. 8). Modulation of miR-10a may effectively block the secretion of IL-1β, TNF-α and IL-6 and act as the neutralizing agent that targets them. Thus, miR-10a acts as a switch to control this regulatory circuit and may be a diagnostic and therapeutic target for RA treatment.

## Methods

### miRNA microarray

For miRNA microarray analysis, we used FLS cells from patients with RA and OA (n = 3 in each group). The patients were all females aged 55–60 years and initially diagnosed with RA or OA. None of the subjects had used immunological drugs or received any treatment. Microarray analysis was performed by KangChen Bio-tech (Shanghai, China) using a miRCURY Hy3 labeling kit (Exiqon, Denmark) and a miRNA array (version 8.1, Exiqon). The detailed methods were performed as follows. Briefly, total RNA was extracted using TRIzol (Invitrogen, Life Technology) and a miRNeasy mini kit (QIAGEN) according to the manufacturer’s instructions. After passing the criteria for RNA quantity measurements performed using a NanoDrop 1000, the samples were labeled using the miRCURY™ Hy3™/Hy5™ Power labeling kit and hybridized to the miRCURY™ LNA Array (v.18.0). Following the washing steps, the slides were scanned using an Axon GenePix 4000B microarray scanner. The scanned images were then imported into GenePix Pro 6.0 software (Axon) for grid alignment and data extraction. A statistical comparison between the RA group and the OA group was carried out to identify differentially expressed miRNAs (Supplementary Fig. S1). The selection criteria involved a two-fold change in the threshold and were significant according to ANOVA (*P* < 0.05).

### Isolation and culture of human RA FLSs

Human synovial tissues were obtained upon arthroplasty or synovectomy from RA or osteoarthritic joints from the Departments of Clinical Immunology and Orthopedics at Xijing Hospital or Tangdu Hospital. All the procedures involving specimens obtained from human subjects were performed under protocols approved by the Human Research Protective Committee of the Fourth Military Medical University and all the methods were carried out in accordance with the guidelines and regulations by the Ethics Committee of the Fourth Military Medical University. Informed consent was also provided by all subjects before initiating the study protocol. RA FLSs were obtained via collagenase digestion as previously described^52^. Briefly, washed synovial tissues without adipose tissue and cartilage were fully diced and transferred into a 1 mg/ml collagenase I solution, followed by a 6-h routine digestion. The liberated cells were then cultured in DMEM supplemented with 10% FBS, 50 U/ml penicillin/streptomycin, and 2 mM L-glutamine. After passaging for three generations, the cells were identified through flow cytometry to form a homogeneous population (phenotype: CD14^−^CD68^−^CD90^+^), and cells in the third to sixth generations were used for subsequent experiments.

### RNA isolation and real-time PCR

Total RNA was extracted from cells with TRIzol, and cDNA was then synthesized with the TaqMan^®^ MicroRNA Reverse Transcription Kit, for miRNA, or the PrimeScript^®^ 1^st^ Strand cDNA Synthesis Kit, for general genes, according to the manufacturers’ instructions. Aliquots of the reaction mixture were used for real-time PCR with TaqMan^®^ 2 × Universal PCR Master Mix under the following conditions: initial denaturation at 95 °C, for 10 min followed by 40 cycles of 95 °C for 15 s, 60 °C for 1 min and 72 °C for 45 sec. The expression of mature miR-10a was normalized to U6 mRNA, and IRAK4, TAK1, BTRC, IL-1β, IL-6, IL-8, MCP-1, MMP1, MMP13 and YY1 were normalized to β-actin mRNA. All PCR experiments were performed in triplicate. The sequences of the primers are listed in Table 1.

### Western blot assay

RA FLSs were completely lysed in ice-cold cell lysis buffer as previously described^52^. Equal amounts of protein corresponding to approximately 40 μg were then subjected to SDS-PAGE electrophoresis and transferred to polyvinylidene difluoride (PVDF) membranes. The membranes were subsequently incubated with IRAK4, TAK1, BTRC, p65, YY1, Histone H3 or β-actin primary antibodies and HRP-conjugated secondary antibodies, and the specific immunoreactive proteins were visualized through enhanced chemiluminescence.

### Chromatin immunoprecipitation (ChIP)

ChIP was carried out according to the manufacturer’s protocol (Millipore, Billerica, MA). Briefly, HEK293T cells transfected with pCMV-YY1 or siYY1 were stimulated with TNF-α for 12 h and fixed with 1% formaldehyde, followed by incubation with modest shaking for 30 min at room temperature. The cells were then washed twice with cold PBS, after which the cell pellets were resuspended and lysed, and nuclei were isolated and sonicated to produce 200–1000 bp DNA fragments. An anti-YY1 antibody and control IgG were used for the ChIP assays. The precipitated DNA fragments were finally quantitated via real-time PCR and normalized against the Input. The sequences of YY1 siRNA (siYY1), the negative control siRNA (siNC) and the primers used for real-time PCR to identify YY1BS1 (BS1, Binding Site 1), and YY1BS2 were listed in Table 1.

### Vector construction and luciferase reporter assays

The dual-luciferase vectors psiCHECK-rcmiR-10a-WT, psiCHECK-IRAK4-3′UTR-WT, psiCHECK-TAK1-3′UTR-WT and psiCHECK-BTRC-3′UTR-WT were constructed by synthesizing the candidate seed sequences in the 3′-UTRs of IRAK4, TAK1, and BTRC, or the reverse complementary sequence of miR-10a (rcmiR-10a), followed by insertion of the annealed products into the psiCHECK-2 vector. For the mutant constructs psiCHECK-rcmiR-10a-MUT, psiCHECK-IRAK4-3′UTR-MUT, psiCHECK-TAK1-3′UTR-MUT and psiCHECK-BTRC-3′UTR- MUT, 3-bp mutations were introduced into the seed sequences (Supplementary Fig. S2). For luciferase reporter assays, HEK-293T cells were seeded in 24-well plates (1.5–3 × 10^6^/well) and transfected with 0.8 μg of the recombinant vectors, either alone or in combination with 30 nM miR-10a precursors or inhibitors. Firefly luciferase (FLuc) and *Renilla* luciferase (*RLuc*) activities were measured in cell lysates 24 h later using the dual-luciferase reporter assay system.

To verify NF-κB regulation of miR-10a through YY1, HEK293T cells were transfected with a pGL-miR-10a-YY1 luciferase reporter vector constructed with the promoter region of miR-10a that contains the two YY1 binding sites, either alone or in combination with the pCMV-p65 or pCMV-YY1 vectors or siYY1. The groups subjected to transfection with pGL-miR-10a-YY1 alone, scramble siRNA (siNC) or the empty vector (pCMV-control) were used as control groups. *Renilla* luciferase activity was measured 24 h later. To further identify the specific effect of YY1 on miR-10a expression, HEK293T cells were transfected with luciferase reporter vectors constructed using the miR-10a promoter region containing mutations in YY1 binding site 1 (pGL-miR-10a-BS1 M) or 2 (pGL-miR-10a-BS1 M), or in both sites (pGL-miR-10a-BS1+2 M); luciferase activity was measured 24 h later.

### Immunofluorescence

FLS cells were fixed in 10% methanol for 10 min. After being washed with PBS three times for 5 min each, the cells were permeabilized with 0.1% Triton X-100 in PBS for 15 min. Then, the nonspecific binding of antibodies was blocked through incubation with 1% bovine serum albumin (BSA) for 1 h, followed by incubation with a rabbit monoclonal primary antibody against p65 at 4 °C overnight. After washing with PBS, the slides were incubated with an Alexa 488-conjugated secondary antibody for 30 min, then counterstained with DAPI for 5 min and assessed via immunofluorescence microscopy (magnification ×80).

### Proliferation assay

FLS cells were stimulated with TNF-α or IL-1β, either alone or in combination with the transfection of miR-10a mimics or a miR-10a inhibitor, and then cultured at 37 °C with 5% CO2 in humid air. Scramble mimics and inhibitor were used as controls. The cells were then seeded in 96-well plates in triplicate (8 × 10^3^/well) 48 h after transfection, as indicated, and cell proliferation was measured with a BrdU assay kit (Roche, Basel, Switzerland) according to the manufacturer’s protocol. The absorbance was measured at 450 nm using a multi-well microplate reader.

### Transwell migration/invasion assay

RA FLSs were starved in serum-poor DMEM for 24 h after transfection for 48 h, and a Transwell assay was then performed using 6.5 mm Transwell chambers with 8-μm pores, as described previously. Briefly, the bottom surface of each membrane was pre-coated with Matrigel. Then, FLSs (5 × 10^4^) were seeded in the upper chambers, and 600 μl of complete medium was added to the lower chambers. After incubation at 37 °C for 24 h, the upper surface of each membrane was cleaned with a cotton swab. Cells that migrated to the bottom side were stained with crystal violet and counted (five fields/well) at 80× magnification under a microscope.

### Wound closure assay

RA FLSs were transfected as indicated, and a wound closure assay was performed to evaluate the ability of the cells to migrate. The cells were trypsinized, counted, and plated at 1 × 10^6^ cells/ml in 12-well dishes. The cells were then incubated to yield a confluent cell layer for wounding. A cell scratch was made with a sterile pipette tip, and the average distance that cells moved from the edge of the scratch towards the center was measured 24 h later.

### Statistical analysis

All data are presented as the mean ± SD from at least three independent experiments and were analyzed with Student’s *t*-test. Differences were considered statistically significant when *P* < 0.05.

## Additional Information

**How to cite this article**: Mu, N. *et al*. A novel NF-κB/YY1/microRNA-10a regulatory circuit in fibroblast-like synoviocytes regulates inflammation in rheumatoid arthritis. *Sci. Rep*. **6**, 20059; doi: 10.1038/srep20059 (2016).

## Supplementary Material

Supplementary Information

## Figures and Tables

**Table 1:** Nucleotide sequences of primers and siRNAs

Gene or product	Primers
β-actin	Forward: 5′- CTGTCCACCTTCCAGCAGATGT-3′
	Reverse: 5′- CGCAACTAAGTCATAGTCCGCC-3′
YY1 (NM_003403)	Forward: 5′- AGAAGAGCGGCAAGAAGAGTT-3′
	Reverse: 5′- CAACCACTGTCTCATGGTCAATA-3′
IRAK-4 (NM_001145258)	Forward: 5′-CCTGACTCCTCAAGTCCAGAA-3′
	Reverse: 5′-ACAGAAATGGGTCGTTCATCAAA-3′
TAK1 (NM_003188)	Forward: 5′-AGCCTGATGACTCGTTGTTG-3′
	Reverse: 5′-TAATGGCTCATCTGCTCCTG-3′
BTRC (NM_003939)	Forward: 5′-CCAGACTCTGCTTAAACCAAGAA-3′
	Reverse: 5′- GGGCACAATCATACTGGAAGTG -3′
MCP-1 (NM_002982)	Forward: 5′-CAGCCAGATGCAATCAATGCC-3′
	Reverse: 5′- TGGAATCCTGAACCCACTTCT -3′
IL-6 (NM_000600)	Forward: 5′-CCCTGAGAAAGGAGACATGTAAC-3′
	Reverse: 5′-CCTCTTTGCTGCTTTCACACATG-3′
IL-8 (NM_000584)	Forward: 5′-TTGGCAGCCTTCCTGATTTC-3′
	Reverse: 5′-TGGCAAAACTGCACCTTCAC-3′
IL-1β (NM_000576)	Forward: 5′- TTCGACACATGGGATAACGAGG-3′
	Reverse: 5′- TTTTTGCTGTGAGTCCCGGAG-3′
MMP-1 (NM_002421)	Forward: 5′-GCTAACAAATACTGGAGGTATGATG-3′
	Reverse: 5′-ATTTTGGGATAACCTGGATCCATAG-3′
MMP-13 (NM_002427)	Forward: 5′- TCCTGATGTGGGTGAATACAATG -3′
	Reverse: 5′- GCCATCGTGAAGTCTGGTAAAAT -3′
siYY1	Sense: 5′-CAGUCAACUAACCUGAAAUTT-3′
	Antisense: 5′-AUUUCAGGUUAGUUGACUGTT-3′
siNC	Sense: 5′-UUCUCCGAACGUGUCACGUTT-3′
	Antisense: 5′-ACGUGACACGUUCGGAGAATT-3′
YY1BS1	Forward: 5′-GGGGAGGTGTGGGGAGG-3′
	Reverse: 5′-AGGTGGCTAGGCGAGTTCATATG-3′
YY1BS1	Forward: 5′-GCTTGGGTTGCTGGTG GTAG-3′
	Reverse: 5′-CCAGACTCTCAGC TTAGTAAATTCACG-3′
