# Supplementary material for: A novel NF-κB/YY1/microRNA-10a regulatory circuit in fibroblast-like synoviocytes regulates inflammation in rheumatoid arthritis
Source: Sci Rep. 2016 Jan 29;6:20059. doi: 10.1038/srep20059 (PMC4731824; doi:10.1038/srep20059)
Supplement: Supplementary Information [file srep20059-s1.doc]

**Title: A novel NF-κB/YY1/microRNA-10a regulatory circuit in fibroblast-like synoviocytes regulates inflammation in rheumatoid arthritis**

Nan Mu^1#^, Jintao Gu^1#^, Tonglie Huang^1#^, Cun Zhang^1^, Zhen Shu^1^, Meng Li^2^, Qiang Hao^1^, Weina Li^1^, Wangqian Zhang^1^, Jinkang Zhao^3^, Yong Zhang^4^, Luyu Huang^5^, Shuning Wang^1^, Xiaohang Jin^6^, Xiaochang Xue^1^*, Wei Zhang^1^*, Yingqi Zhang^1^*

^1^State Key Laboratory of Cancer Biology, Department of Biopharmaceutics, School of Pharmacy, Fourth Military Medical University, Xi’an, China, ^2^Department of Pharmacogenomics, School of Pharmacy, Fourth Military Medical University, Xi’an, China, ^3^Department of Clinical Immunology and Rheumatology, Xijing Hospital, Fourth Military Medical University, Xi'an, China, ^4^Institute of Orthopedics, Tangdu Hospital, Fourth Military Medical University, Xi'an, China, ^5^Department of Orthopedics, Xijing Hospital, Fourth Military Medical University, Xi'an, China, ^6^Department of Human Anatomy, Histology, and Embryology, Fourth Military Medical University, Xi'an, China.

*Correspondence and requests for materials should be addressed to X.X. (xue_xiaochang@yahoo.com, W.Z. (zhangw90@fmmu.edu.cn) or Y.Z. (zhangyqh@fmmu.edu.cn)

^#^These authors contributed equally to this work.

**Supplementary information**

**Reagents**

DMEM, FBS, penicillin/streptomycin, Lipofectamine 2000 and Trizol were purchased from Life Technologies (Carlsbad, CA). TNF-α and IL-1β were obtained from Peprotech (Rocky Hill, NJ). Bay11-7082, CHX, DAPI and Collagenase I were procured from Sigma Aldrich (St. Louis, MO); SP600125 and SB203580 were procured from Tocris Bioscience (Bristol, UK). The monoclonal antibodies against IRAK4, BTRC, p65 and H3 were purchased from Cell Signaling Technology (Danvers, MA), those against TAK1 were procured from Epitomics (Burlingame, CA), and those against YY1 and β-actin were obtained from Abcam (Cambridge, MA). HRP-conjugated secondary antibodies were purchased from CWBIO (Beijing, China). Restriction endonucleases and T4 DNA ligase were procured from New England Biolabs (Ipswich, MA). The psiCHECK-2 vector and dual-luciferase reporter system were obtained from Promega (Madison, WI). The miR-10a and U6B PCR primers, miR-10a precursors (mimics) and inhibitors, and scramble mimics and inhibitors were purchased from Ambion (Carlsbad, CA). The TaqMan® microRNA reverse transcription kit and gene expression assays were obtained from Applied Biosystems (Carlsbad, CA). The chromatin immunoprecipitation kit and transwell chamber were purchased from Millipore (Billerica, MA). Matrigel was procured from BD Biosciences (San Diego, CA). The cell proliferation ELISA and BrdU (Colorimetric) were obtained from Roche (Basel, Switzerland). The BCA protein assay kit and the nuclear and cytoplasmic extraction reagents were procured from Thermo Scientific (Rockford, MN). Human IL-6 and IL-8 ELISA kits were purchased from Cusabio (Beijing, China). Nucleotides were synthesized by AuGCT Biotechnology Co., Ltd. (Beijing, China).


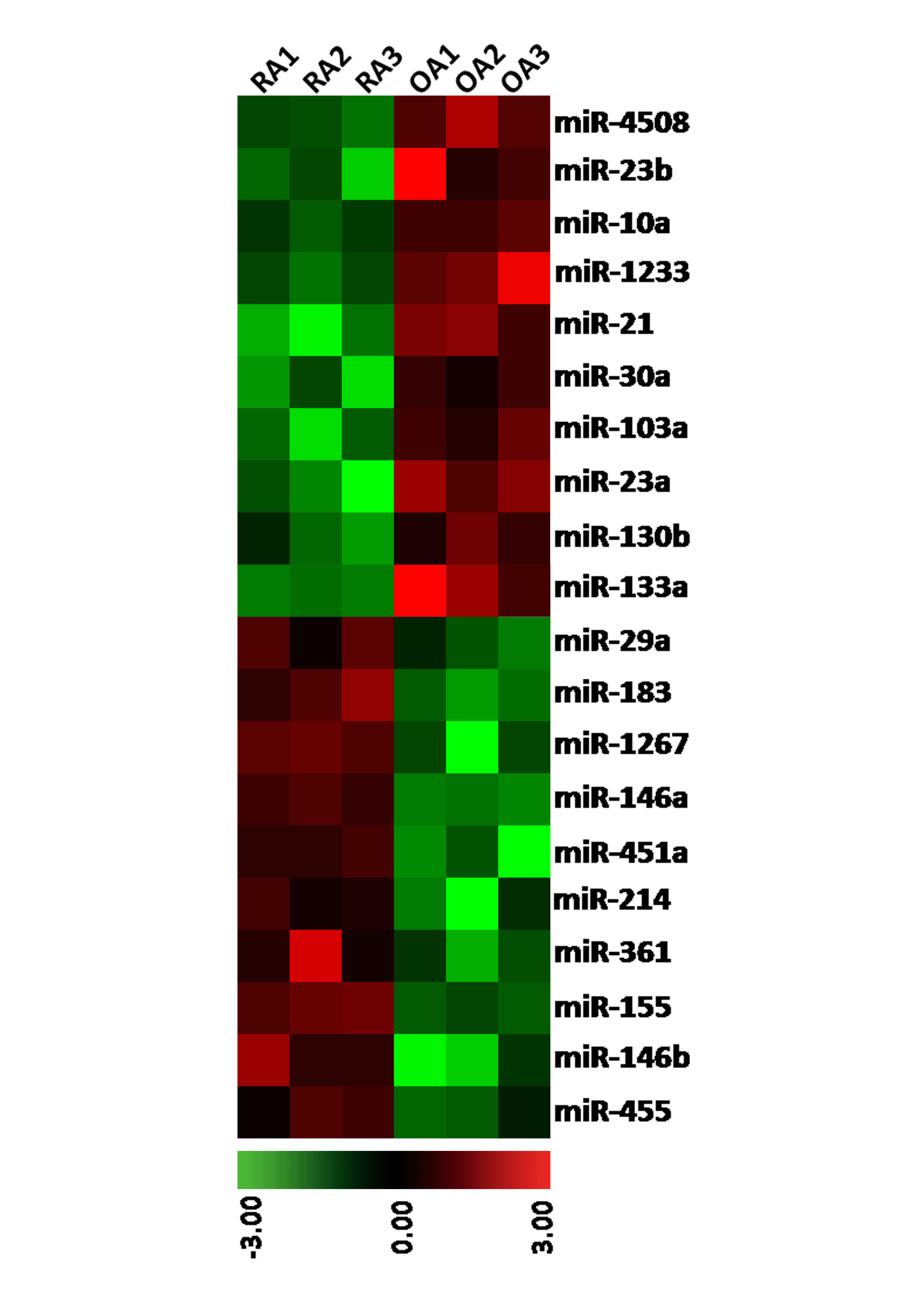


**Figure S1 ׀ The top ten differentially expressed miRNAs in RA FLSs compared with OA FLSs.** Total RNA was extracted from FLS cells of RA and OA patients (n = 3 in each group) and microarray analysis was performed by KangChen Bio-tech (Shanghai, China) according to the protocol described in Method. A statistical comparison between the RA group and the OA group was carried out to identify differentially expressed miRNAs.


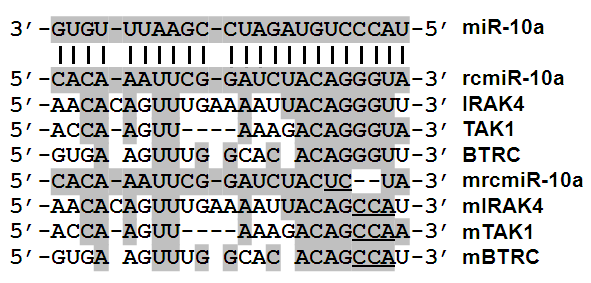


**Figure S2 ׀ Sequence alignment of miR-10a with the 3’-UTRs of target genes.** Alignment of the miR-10a sequences with the reverse complimentary miR-10a (rcmiR-10a) and 3’-UTR of IRAK4, TAK1 and BTRC, or with the corresponding sequences containing mutations in the seed sequence (mrcmiR-10a, mIRAK4, mTAK1 and mBTRC); mutated nucleotides are underlined.


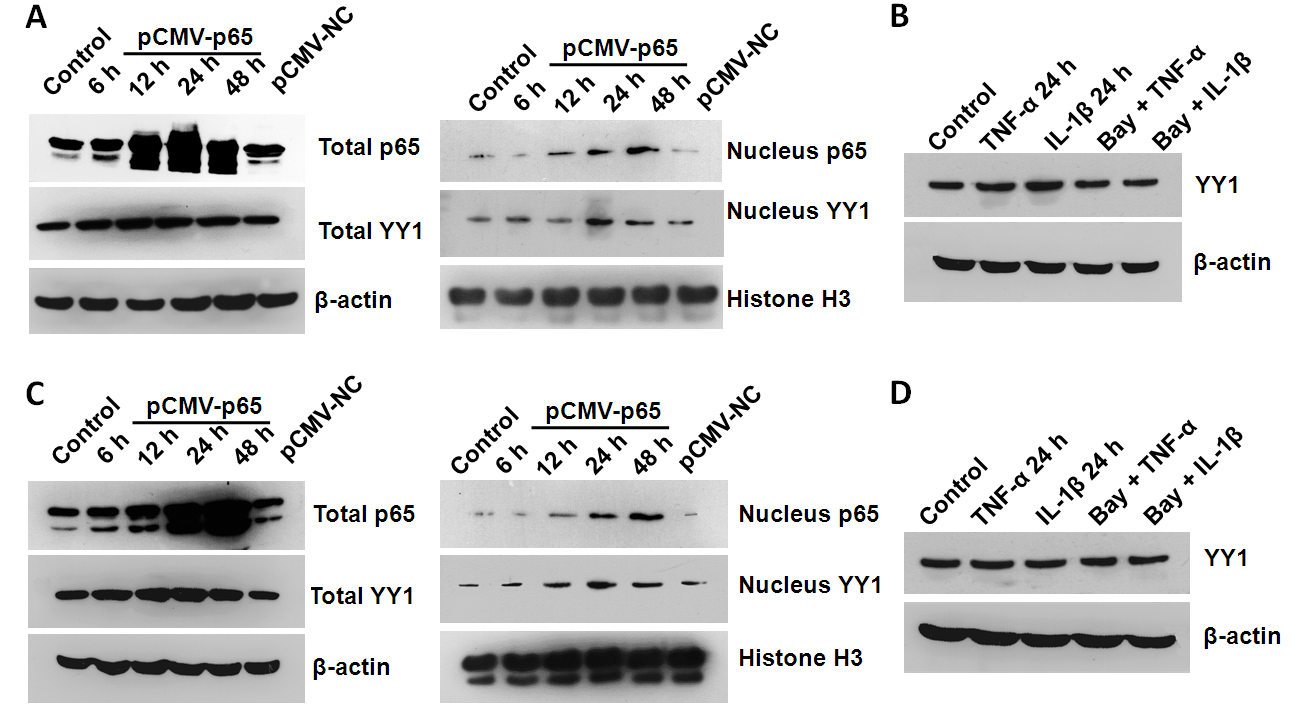


**Figure S3 ׀ TNF-α and IL-1β or activated p65 promotes YY1 expression and translocation.** **(A, C)** HEK293T cells were transfected with pCMV-p65, and the expression levels of p65 and YY1 were detected by Western blotting. **(B, D)** RA FLSs were stimulated with TNF-α or IL-1β, either alone or in combination with Bay 11-7082 for 24 h, and the YY1 level was detected by Western blotting. The data are representative of three experiments (Twice of the data, the other one is shown in the manuscript).
